# Cathelicidin LL-37 in Health and Diseases of the Oral Cavity

**DOI:** 10.3390/biomedicines10051086

**Published:** 2022-05-07

**Authors:** Joanna Tokajuk, Piotr Deptuła, Ewelina Piktel, Tamara Daniluk, Sylwia Chmielewska, Tomasz Wollny, Przemysław Wolak, Krzysztof Fiedoruk, Robert Bucki

**Affiliations:** 1 Department of Medical Microbiology and Nanobiomedical Engineering, Medical University of Białystok, Mickiewicza 2C, 15-222 Białystok, Poland; asiatokajuk@gmail.com (J.T.); piotr.deptula@umb.edu.pl (P.D.); tamara.daniluk@umb.edu.pl (T.D.); sylwia.chmielewska@umb.edu.pl (S.C.); krzysztof.fiedoruk@umb.edu.pl (K.F.); 2 Dentistry and Medicine Tokajuk, Zelazna 9/7, 15-297 Bialystok, Poland; 3 Independent Laboratory of Nanomedicine, Medical University of Białystok, Mickiewicza 2B, 15-222 Białystok, Poland; ewelina.piktel@wp.pl; 4 Holy Cross Oncology Center of Kielce, Artwińskiego 3, 25-734 Kielce, Poland; tomwollny@gmail.com; 5 Institute of Medical Science, Collegium Medicum, Jan Kochanowski University of Kielce, IX Wieków Kielc 19A, 25-317 Kielce, Poland; przemyslaw.wolak@ujk.edu.pl

**Keywords:** oral cavity, human cathelicidin, antimicrobial peptides, immunomodulation

## Abstract

The mechanisms for maintaining oral cavity homeostasis are subject to the constant influence of many environmental factors, including various chemicals and microorganisms. Most of them act directly on the oral mucosa, which is the mechanical and immune barrier of the oral cavity, and such interaction might lead to the development of various oral pathologies and systemic diseases. Two important players in maintaining oral health or developing oral pathology are the oral microbiota and various immune molecules that are involved in controlling its quantitative and qualitative composition. The LL-37 peptide is an important molecule that upon release from human cathelicidin (hCAP-18) can directly perform antimicrobial action after insertion into surface structures of microorganisms and immunomodulatory function as an agonist of different cell membrane receptors. Oral LL-37 expression is an important factor in oral homeostasis that maintains the physiological microbiota but is also involved in the development of oral dysbiosis, infectious diseases (including viral, bacterial, and fungal infections), autoimmune diseases, and oral carcinomas. This peptide has also been proposed as a marker of inflammation severity and treatment outcome.

## 1. Introduction

The regular flow of nutrients in the temperature-stable and hydrated environment of the oral cavity, along with its spatial diversity, creates a perfect ecological habitat for various microorganisms, which shortly after the birth of the host become their integral part as the oral microbiota, or more broadly, microbiome (i.e., bacterio-, archae-, myco-, viro-, and protozoome) [1]. In normal healthy conditions, these highly biodiversified microbial populations, represented by ~1000 species, live in metabolically cooperative and self-regulating, e.g., via *quorum sensing*, biofilm communities coexisting in a symbiotic/commensal relationship with the human host (in a eubiosis state) and due to their colonization resistance role, are considered as a part of a nonspecific defense system. Therefore, the term “oralome” has been proposed to encompass all inter-microbial as well as host–microbiome interactions underlying the oral cavity ecosystem [1]. However, any disturbance of this homeostasis, resulting in uncontrolled overgrowth of certain bacterial species (dysbiosis or “unbalanced microbiome”) and, in turn, their transition from a commensal to parasitic status, is responsible for the development of pathological conditions such as infectious and autoimmune diseases, along with the possible longitudinal consequences, such as oral carcinomas [2]. The most common oral cavity diseases associated with microorganism-induced inflammation include: dental caries, pulpitis, refractory apical periodontitis, periodontal diseases, implant-associated infection, candidiasis, and diseases of the oral mucosa. Yet, the pathogenesis of these remarkably polymicrobial and successively progressing diseases is known in a general outline only. Although the major implicated bacterial species are identified and classified into six color-coded complexes according to the role (the Socransky complexes) [3,4], underlying host factors, in particular immunity, are poorly understood. Briefly, the members of yellow, green, and purple complexes (e.g., *Streptococcus, Veilonella,* and *Actinomyces*) are considered as the primary or early colonizers of the oral cavity, which allow the succession of the secondary, the orange complex, e.g., *Fusobacterium nucleatum*, which acts a “bridge” for the late, red complex, colonizers. It is believed that the increased proportion of the latter two, in particular the red one, involving three obligate anerobic Gram-negative species, namely *Porphyromonas gingivalis*, *Tannerella forsythia*, and *Treponema denticola*, considered as the major periodontal pathogens, leads to pathological, associated with excessive immune response, changes resulting in gingival connective tissue and alveolar bone damage manifested clinically as periodontal diseases [3]. Hence, the distinction between these “good” (or beneficial) and “bad” (or useless/pathogenic) commensals may allow the development of new therapies for certain oral diseases. Specifically, their imbalanced proportion must be reconciled with nonspecific, i.e., the relying on microbial-associated molecular patterns (MAMPs) and pattern recognition receptors (PRRs), nature of the innate response and implicates a fine-tuned strategy of its coordination, e.g., via positive/negative feedback loops with the adaptive immune system, to appropriately respond to qualitative and quantitative changes in the composition of oral microbiota. Endogenous antimicrobial peptides (AMPs), such as cathelicidins or defensins, appear to be vital elements of this strategy [5,6,7]. In fact, the functional versatility and structural diversity—represented by over 100 various human AMPs—suggest that the protection of a specific body’s microenvironments, rather than the systemic action, as well as maintaining homeostasis with microbiota, are the major function of these small, <100 amino acids, peptides [8]. For instance, over 45 AMPs are found in the human saliva and the gingival crevicular fluid (GCF), which are classified into six major functional classes, namely (i) cationic peptides (CAMPs), (ii) mediating bacterial agglutination and adhesion, (iii) metal ion chelators, (iv) peroxidases, (v) protease inhibitors, and (vi) peptides, with activity against bacterial cell walls [5,6]. Indeed, in the recent literature review by Silva et al. focused on AMPs in controlling the oral pathogens [9], the cathelicidin LL-37 and β-defensin-2 are two AMPs most commonly linked with the periodontal and cariogenic pathogens, respectively. Similarly, the elimination of CAMPs from human airway fluid, via a cation-exchange chromatography, was correlated with a substantial reduction in its antibacterial activity [10].

Hence, the term “host defense peptides” (HDPs) better reflects their role [11]. However, over a decade after the legitimation of their importance for oral health stability by the Seventh European Workshop on Periodontology [12], we are still far from understanding the exact role of these immensely pleiotropic and diverse peptides in this process. For instance, it is believed that the microenvironment-specific action is mediated via different, synergistically acting, combinations (or “cocktails”) of AMPs, allowing efficient elimination of the target pathogens and/or their toxic products at concentrations not affecting the host cells and/or commensal microbes [13]. Indeed, the expression of AMPs is unevenly regulated by various bacterial species, including periodontal and oral mucosa microorganism(s). Thus, the composition of AMP cocktails may reflect the physiological response to specific pathogen(s). It is consistent with the observation that PRRs, such as Toll-like (TLRs) and NOD-like (NLRs) receptors, of oral epithelial cells may induce antibacterial actions, e.g., β-defensins production without concomitant inflammatory response, or the secretion of pro-inflammatory cytokines, such as interleukin-8 (IL-8) [14,15]. Accordingly, a mechanism where AMP-resistant, “good” commensal bacteria induce their expression to control “bad” or potentially pathogenic species has been proposed as a form of adaptive mutualistic co-evolution between the oral microbiota and the host [16].

Conversely, AMPs may act as natural buffers neutralizing the pro-inflammatory stimuli, e.g., LPS or (lipo)teichoic acids, produced by commensal bacteria in order to maintain the oral microbiota–tissues equilibrium. This hypothesis is supported by the observation that the concentrations of most AMPs found in body fluids, e.g., in saliva or GCF, do not reach minimum inhibitory concentration (MIC) values recorded by in vitro tests. Hence, biological activities other than bactericidal are fulfilled by AMPs [14,15]. In fact, many AMPs are classified as damage-associated molecular patterns (DAMPs), i.e., molecules released from damaged or dying cells, and initiate a diverse range of physiological and pathophysiological functions, acting as “alarmins” in the immune surveillance system [14,15]. Moreover, to make it more complicated, the recent metagenomic studies indicate the existence of populational variations in the oral microbiota composition, the so-called stomatotypes, characterized, for example, by the predominance of Proteobacteria (stomatotype 1) and Bacteroides (stomatotype 2) in the study by Willis et al. [2].

**Figure 1:**
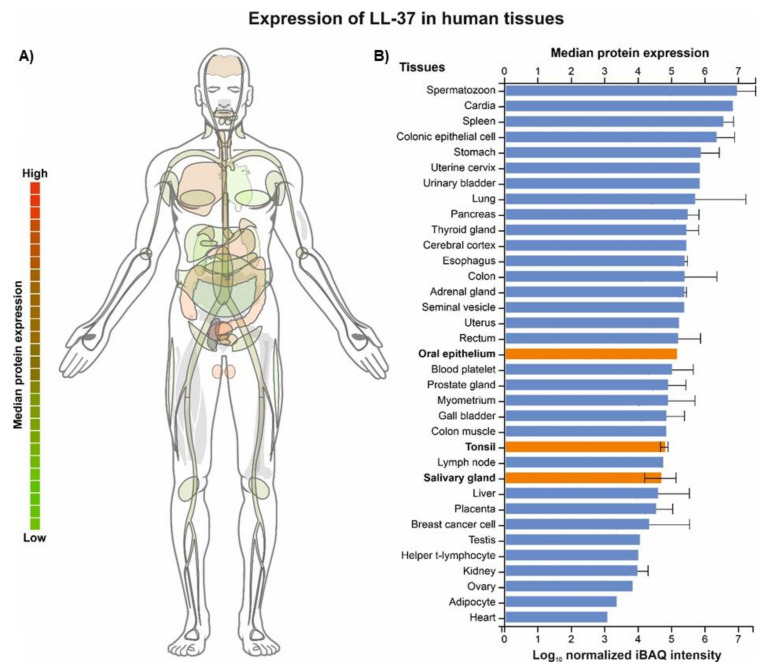
Expression of LL-37 in 35 human tissues, including oral epithelium, tonsils, and salivary glands (highlighted in orange). The data were obtained from ProteomicsDB (https://www.ProteomicsDB.org) last accessed 25 March 2022 [23] and represent mass spectrometry (MS1-level) proteome quantification; the intensity-based absolute quantification (iBAQ) value is a measure of protein abundance and corresponds to the sum of all the peptide intensities divided by the number of observable peptides of a protein (panel **A**). Relative abundance of CAMPs, including LL-37 (highlighted in red), present in the oral cavity (whole organism—integrated; LL-37 is highlighted in red). The data were obtained from PAXdb (Protein Abundance Database; https://pax-db.org/) last accessed 25 March 2022 ) [24] and are based on a mass-spectrometry-based study of the human proteome by Wilhelm et al. (2014) [25]; parts-per-million (ppm) values represent the abundance of each protein with reference to the entire expressed proteome, i.e., each protein entity is enumerated relative to all other protein molecules in the sample (panel **B**).

A compelling amount of evidence demonstrates that the LL-37 peptide is one of the AMPs that appear to be vital for maintaining the eubiosis in the oral cavity and, hence, it may serve as a potential agent for pharmaceutical prophylaxis or the treatment of its various dysbiotic conditions, e.g., through direct application, the application of its synthetic derivatives, or the modulation of its expression [17,18]. In fact, the therapeutic potential of LL-37 in other diseases has been already recognized by biopharmaceutical companies, e.g., in the treatment of venous leg ulcers (Promore Pharma AB, Sweden) [19] or chronic suppurative otitis media (OctoPlus BV) [20]. Currently, 69 clinical trials are recorded in the ClinicalTrials database (www.clinicaltrials.gov; search term: LL-37 or cathelicidin; accessed on 25 March 2022) evaluating its role and therapeutic/diagnostic potential in >30 different diseases/disorders, ranging from (i) bacterial infections, sepsis, periodontitis, tuberculosis, and HIV to (ii) various skin (e.g., psoriasis and atopic dermatitis), intestinal tract (e.g., Crohn disease), or respiratory tract conditions (e.g., asthma and cystic fibrosis) and (iii) melanoma, chronic kidney disease, and vitamin D deficiency. In particular, the role of vitamin D, as a potent cathelicidin inducer, is frequently studied in this context. However, due to a wide distribution and abundance of LL-37 across the human body (Figure 1), as well as its association with the pathogenesis of several infectious and non-infectious diseases [21,22], it seems to be relevant to consider potential medical and safety implications of LL-37-based therapies.

In the current review, we discuss possible mechanisms behind the protective role of LL-37 in the oral cavity, supplementing the literature with data from the constantly growing “-omics” and “-ome,” databases e.g., genomic, proteomic, transcriptomic, interactome, or reactome databases, as a source of comprehensive information about protein expression, distribution, and interaction networks in the human body.

## 2. Mechanism of LL-37 Expression in the Oral Cavity and Its Action at the Molecular Level

Perceiving the human cathelicidin, encoded by the CAMP gene (cathelicidin antimicrobial peptide, i.e., containing the cathelin-like domain), as a single protein is oversimplification. In fact, due to its biological functional diversity, directed by multiple size variants and concentration- or microenvironment-dependent action, LL-37 is synonymous with “versatile,” “pleiotropic,” “multifunctional,” “multifaceted,” “factotum,” or even “moonlighting” protein [18,21,26,27]. Although, the latter term is a misnomer, since cathelicidin does not meet the condition of no post-translational modifications, it shares the dark side of protein moonlighting, namely implication in various diseases, including cancers [28].

**Figure 2:**
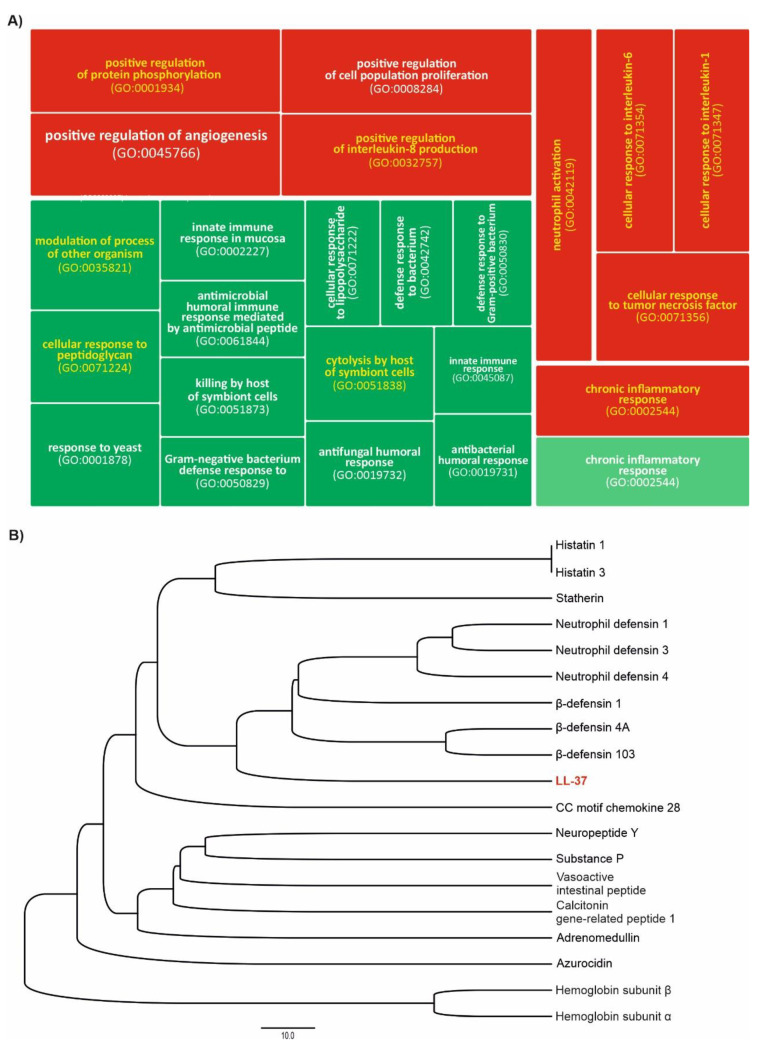
Tree map of the Biological Process (BP) Gene Ontology (GO) category for human cathelicidin LL-37 (panel **A**); green and red boxes show processes associated with immune responses against microorganisms and immunomodulatory action, respectively. Functional similarity of 20 human cationic antimicrobial peptides (CAMPs) present in the oral cavity inferred from comparing their Biological Process (BP) Gene Ontology (GO) category (panel **B**); the BPs unique for human cathelicidin LL-37 are indicated by yellow color in panel A. The dendrogram was constructed using Dice coefficient and UPGMA clustering with NTSYSpc software ver. 2.1 (Exeter Software) based on the GOs collected from the QuickGO server (www.ebi.ac.uk/QuickGO/).

According to the Gene Ontology (GO) database, the CAMP gene is implicated in 24 biological processes (BPs), of which 16 are associated with immune responses against microorganisms, e.g., cell lysis and cellular response to LPS or peptidoglycan, whereas the remaining 9 represent various immune modulatory functions (both pro- and anti-inflammatory), e.g., neutrophil activation, cellular response to interleukins, chronic inflammatory response, or angiogenesis regulation (Figure 2A). These immunomodulatory actions are exerted by LL-37 interaction with various cellular receptors, e.g., chemotaxis (angiogenesis and wound healing) via the formyl receptor-like 1 (FPRL1) or alternatively by the CXC chemokine receptor 2 (CXCR2), cytokine release via the purinergic receptor R2X7, and IL-8 release in the lung epithelial cells through the epidermal growth factor receptor (EGFR), as reviewed by Verjans et al. [29].

**Figure 3:**
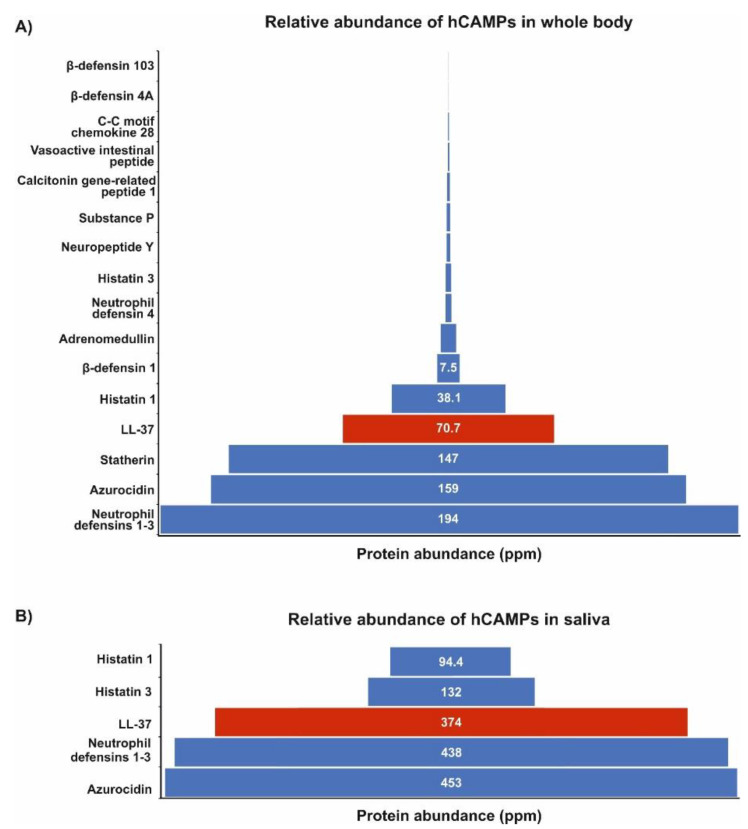
Relative abundance of CAMPs, including LL-37 (highlighted in red), in whole organism (panel **A**) and saliva (panel **B**). The data were obtained from PAXdb (Protein Abundance Database; https://pax-db.org/, accessed on 25 March 2022) [24] and are based on a mass-spectrometry-based study of the human proteome by Wilhelm et al. (2014) [25]; parts-per-million (ppm) values represent the abundance of each protein with reference to the entire expressed proteome, i.e., each protein entity is enumerated relative to all other protein molecules in the sample.

**Figure 4:**
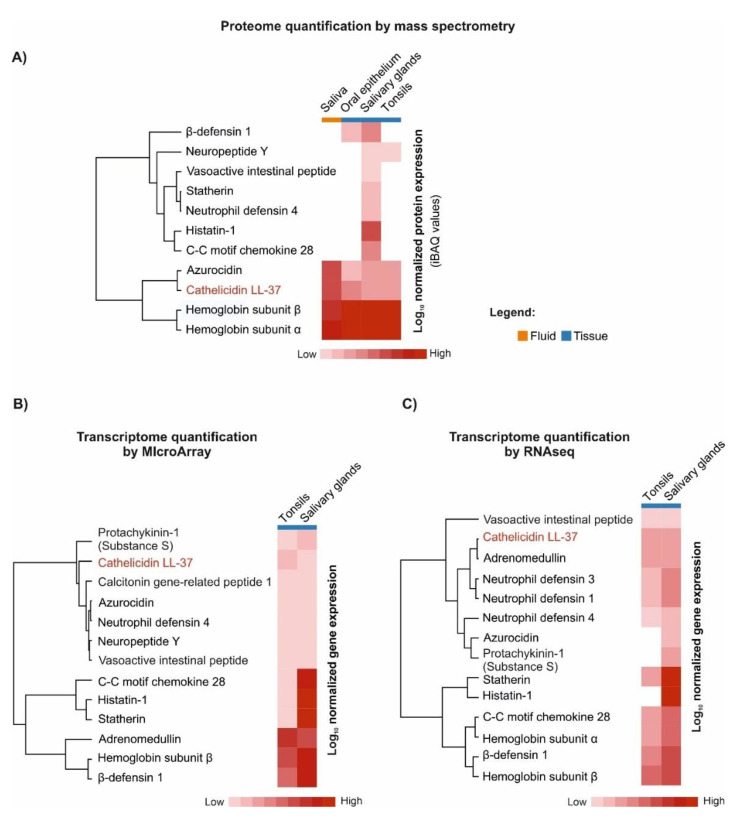
Comparison of CAMPs expression of in saliva, oral epithelium, tonsils and salivary glands. The data were obtained from ProteomicsDB (https://www.proteomicsDB.org, accessed on 25 March 2022) [23], and represent mass spectrometry (MS1-level) human proteome quantification; the iBAQ (intensity-based absolute quantification) value is a measure of protein abundance, and corresponds to the sum of all the peptides intensities divided by the number of observable peptides of a protein (panel **A**). Correspondingly, the gene expression results inferred from MicroArray and RNAseq transcriptomic studies are shown in panel **B** and **C**, respectively.

Remarkably, in the “immunomodulatory” group, 7 categories are unique for LL-37, in comparison to 19 other AMPs from the oral cavity, revealing its extraordinary potential to modulate the immune response and, in general, its functional dissimilarity (Figure 2B). Furthermore, according to the data from the Protein Abundance Database (PAXdb), among these AMPs, LL-37 is the fourth- and third-most-abundant AMP in the whole body and saliva, respectively (Figure 3). This relative abundance of LL-37 in the oral cavity is supported also by other proteomic and transcriptomic data (Figure 4). It is noteworthy that in contrast to the microbicidal activity, immunomodulatory functions of LL-37, such as anti-endotoxic, wound-healing, or angiogenic ones, are unaffected by the physiological salt concentrations and, in general, are exerted at low concentrations (<1 μM) [27].

**Figure 5:**
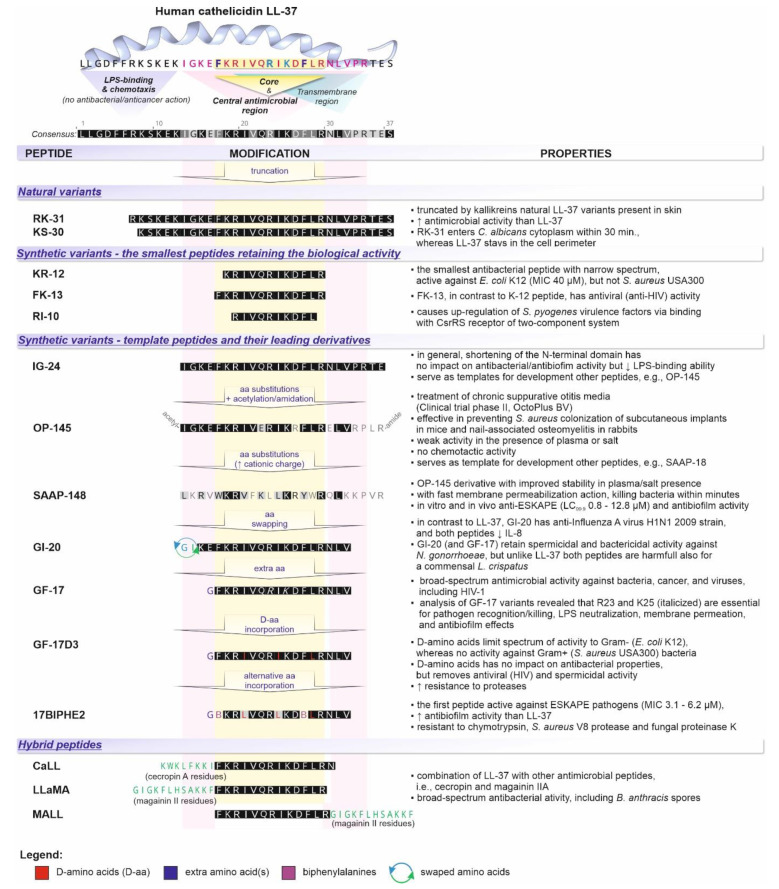
LL-37 and its selected natural and synthetic variants. Amino acids identical to the residues in native LL-37 are illustrated on a black background (with white letters); the others represent substitutions and/or modifications. The core antimicrobial region LL-37 residues essential for its interaction with cell membranes, i.e., phenylalanines F17/F27, and pathogen recognition/killing; LPS neutralization; membrane permeation; and antibiofilm effects, i.e., arginine—R23 and lysine—K25, are in bold and highlighted in violet and green, respectively.

Furthermore, both activities are mediated by distinct regions of the LL-37 peptide; for example, amino acid residues 13–32 and 17–29 are a part of the central and core antimicrobial region, respectively. The first twelve N-terminal residues are crucial for chemotaxis and LPS neutralization, although in the latter cases, both N- and C-terminal residues are relevant (Figure 5). The possibility to separate both activities, e.g., toward microbicidal or immunomodulatory, via amino acid sequence manipulation, illustrates the therapeutic potential of LL-37 (see below). Correspondingly, although the LL-37 peptide—the product of proteolytic cleavage of 18 kDa cathelicidin precursor protein (hCAP-18) by proteinase-3 in neutrophils—is, in general, synonymous with the human cathelicidin, depending on the hCAP-18 processing proteases, other variants can be distinguished. For instance, in an acidic vaginal environment, gastricsin cleaves hCAP-18 into ALL-38 peptide, and TLN-58 variant, found in the lesion vesicle of palmoplantar pustulosis, is a product of skin proteases (kallikreins) [30]. In addition, the latter proteases can split the mature LL-37 into several peptides, such as RK-31, KR-30, KS-20, KS-22, KS-27, or LL-29, characterized by the same or even superior (RK-31 and KS-30) antimicrobial, but attenuated immunomodulatory, action than the native LL-37 (Figure 5) [31].

**Figure 6:**
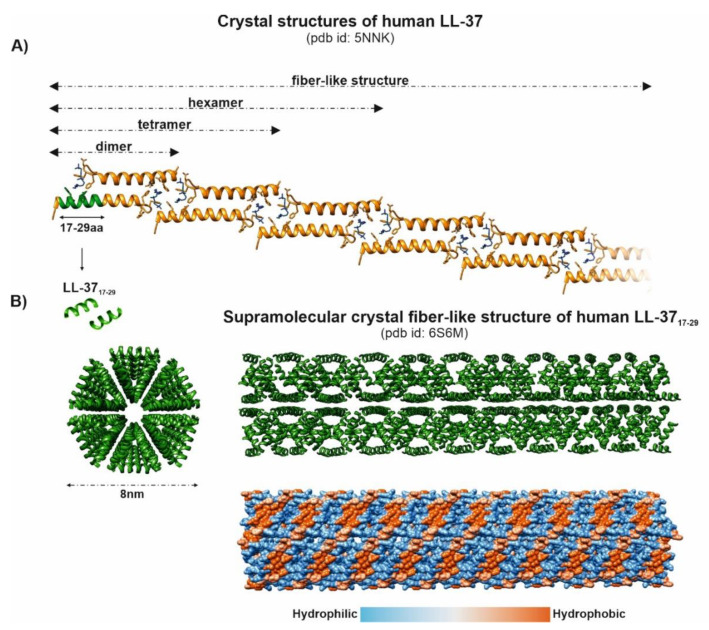
Human LL-37 oligomer structures interacting with lipid membranes (panel **A**) and its antimicrobial core fragment (residues 17–29), LL-37_17-29_, forming supramolecular fiber-like structures (panel **B**), described by Sancho-Vaello et al. [38] and Engelberg and Landau [36], respectively.

Structurally, LL-37 is an amphipathic peptide with two helical regions separated by a loop and a short unstructured C-terminal tail [32] (Figure 5). It was reported that the net charge +6 directs LL-37 activity toward negatively charged bacterial surfaces, rather than human cells, leading to higher values of cytotoxic concentrations for eukaryotic cells (1–10 μM vs. 13–25 μM) [33]. In the physiological salt concentrations, ~35% content of hydrophobic residues allows LL-37 to adopt an amphipathic helical structure, which is further stabilized by interaction with cell membranes. However, the exact mechanisms of membrane permeation by LL-37 and its selectivity toward microbial membranes are still debated [33,34]. Remarkably, the recent studies in this area have revealed impressive conformational plasticity of LL-37 and its truncated variants, reflected by the formation of various oligomeric and supramolecular fiber-like structures (Figure 6). Importantly, such LL-37 fibers appear to affect cell membranes differentially than classical “barrel-stave” or “carpet/toroidal”-type mechanisms [35,36] (Figure 6). For example, Sancho-Vaello et al. proposed a model of pore formation via LPS extraction from the outer membrane by LL-37, with its subsequent diffusion into the periplasmic space, followed by interaction with the inner membrane involving oligomerization and fiber formation [37]. Furthermore, the authors identified a single arginine residue (Arg_23_) as crucial in “eukaryotic” lipid, i.e., saturated lipid, recognition and suggested that the LL-37-fibrils mediated the recruitment of the host lipids as a novel, “armor-like,” mechanism of antibacterial defense [37,38]. Similarly, depending on the lipid composition, Shahmiri et al. described two distinct LL-37 membrane interaction pathways, characterized by (i) pore formation in bilayers of unsaturated phospholipids and (ii) membrane modulation with saturated phospholipids, followed by the formation of fibrous peptide-lipid superstructures in the latter pathway [33]. Finally, LL-37 fibrils, via binding with DNA and forming spatially periodic DNA nanocrystalline immunocomplexes, have been described as potent simulators of the TLR-9-mediated response [39]. In general, binding extracellular DNA/RNA is a basic function of LL-37 that stabilizes nucleic acids and prevents degradation by the host and bacterial DNases/RNases, as well as enhances their uptake by various cells, such as macrophages, dendritic cells, and B cells, leading to TLR9 and TLR7 activation [40]. The same function of LL-37 is observed in neutrophil extracellular traps (NETs) [41]. However, at an antimicrobial and inflammatory focus, citrullination of its arginine residues, catalyzed by peptidyl-arginine deiminases, compromises the bactericidal activity of LL-37 and abrogates its TLR signaling and, hence, immunomodulatory functions [42]. However, citrullination of LL-37, and in turn impairing pro-inflammatory responses in macrophages, may be a potential treatment method of sepsis [13].

Assuming antimicrobial activity, it should be noted that LL-37 has a unique, among helical AMPs, ability to produce a helical structure in the absence of cell membranes. Therefore, LL-37 is prone to oligomerization or binding with other hydrophobic molecules, which in turn decreases its membrane permeation potential and explains a lower antimicrobial activity in the presence of body fluids, e.g., saliva, plasma, or rich media [27,43]. The antimicrobial activity of LL-37 is also reduced (2- to 8-fold) in the presence of 100 mM Na^+^, as well as at low pH values; at the same time, saliva provides protection against LL-37 degradation by proteases secreted by periodontal pathogens [44]. Additionally, concentrations of LL-37 in saliva (0.14–3 μg/mL) [5] and GCF (0.01–10.8 μg/mL) [45] are lower than the MIC for periodontal pathogens, suggesting existing in vivo higher local concentrations of LL-37 or that other biological functions are important in the oral cavity [5]. However, laboratory susceptibility tests may not reflect in vivo situations. For instance, bacteria in the presence of carbonate, e.g., in saliva, are more susceptible to LL-37, and likewise in hypoxic conditions [5,6]. Therefore, the direct antimicrobial killing action of LL-37, alone or in combination with other AMPs, may be an important mechanism of defense in oxygen-deficient environments, such as inflamed gingiva, where O_2_-dependent bactericidal activity of phagocytes is reduced [5,6]. Accordingly, Wuersching et al. have recently reported that O_2_ availability alters the antimicrobial activity of LL-37 [46] (see below). Conversely, the major direct antimicrobial action of LL-37 may be the prevention of biofilms formation, which occurs at subinhibitory concentrations as low as 0.5 µg/mL, and represents 1/128 of the MIC value [47].

Nevertheless, controlling of the intensity of the immune response induced by LPS/LTA, flagellin, and other molecules released by the oral microbiota (MAMPs) occurs in the physiological range of LL-37 concentrations (1–5 µg/mL) and appears to be a fundamental protective function of LL-37 [48]. Several studies have reported dismissing by LL-37 of various LPS-induced inflammatory responses, including protection against periodontal bone resorption [49], via blocking the binding of LPS to CD14 and lipopolysaccharide-binding protein (LBP) and/or indirect effects on immune cells [50]. In general, modulation of inflammation by LL-37 in response to the bacterial challenge is mediated through balancing TLR activation [40,51]. For instance, bacteria killed by LL-37 or the addition of LL-37 to non-viable bacteria strongly inhibits TLR activation. This “silent” killing reduces the inflammatory response and, in turn, tissue damage by unnecessary inflammation when the bacteria are no longer harmful [40]. It is also crucial from the perspective of concentration-dependent activity of LL-37.

Since the expression of LL-37 is not directly regulated by LPS and other MAMPs but is TLR dependent, it can be affected by other factors that directly coordinate the CAMP gene expression, thereby affecting its ultimate antimicrobial action. Vitamin D metabolite 1,25-dihydroxyvitamin D3 (1,25(OH)D3) has been recognized as the most important inducer of LL-37 expression, through binding with so-called vitamin D response elements (VDRE) for the vitamin D receptor (VDR) in the CAMP gene promotor. In fact, vitamin D links LL-37 expression with MAMPs or other inflammatory stimuli because the expression of 1-α-hydroxylase (CYP27B1), which converts 25-hydroxyvitamin D3 to the active 1,25(OH)D3, can be also upregulated by the stimulation of TLRs [52]. Conversely, 1,25(OH)2D3 has no impact on LL-37 production in colonic epithelial cells, where butyrate acts as the CAMP gene inducer. Likewise, TNF-α, IL-17A, TLR agonists, insulin-like growth factor, MUC2 mucin, simvastatin, injury and wounding, and endoplasmic reticulum stress upregulate LL-37 expression. Certain bacteria (*Shigella* and *Neisseria*)*,* bacterial exotoxins, IFN-γ, IL-6, glucocorticoids, transmigration across activated endothelium, and calcipotriol have been identified as downregulating agents [22,27].

Accordingly, McMahon et al. have shown that 1,25(OH)2D3 can induce not only the expression of LL-37 but also the innate immune regulator TREM-1 (triggering receptor expressed on myeloid cells), which augments TLR-mediated antimicrobial responses and the production of proinflammatory chemokines and cytokines in response to bacterial and fungal infections [53].

## 3. Involvement of the LL-37 Peptide in Maintaining Homeostasis of Oral Microbiota

**Figure 7:**
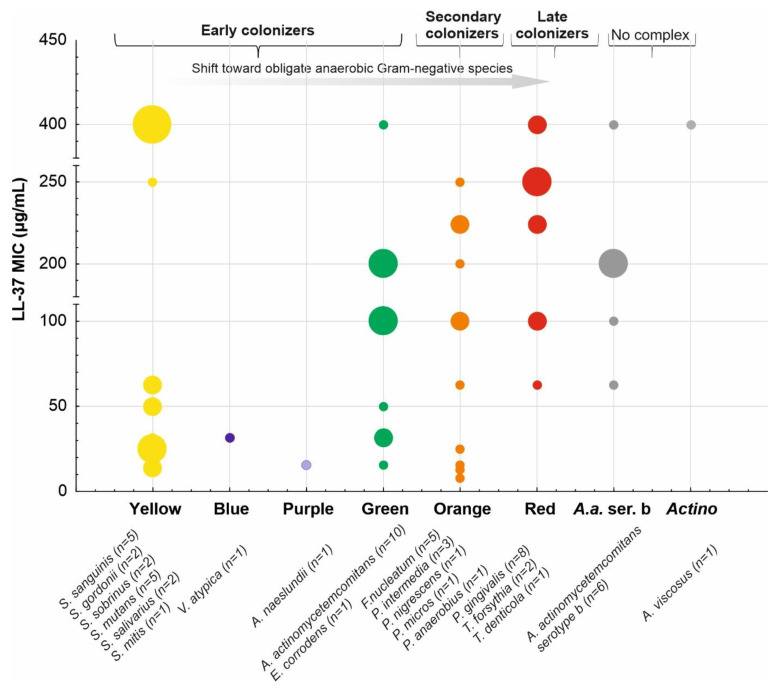
Susceptibility of selected bacteria representing the periodontal complexes (yellow, blue, purple, green, orange, and not-belonging-to-any-complex *A. actinomycetemcomitans* serotype b and *A. viscosus*) to LL-37 (MIC values, μg/mL) based on the references [55,57,58,59,60]; the repeated strains in various studies were analyzed individually, and for MICs expressed as “>” or not recorded “≤,” for example, >100 or ≤100, the higher dilution value, i.e., 200, was used.

Resistance to killing and/or evasion of the host immune responses is an inherent characteristic of pathogenic microorganisms that discriminates them from commensal species. Accordingly, to recognize the role of immune response in periodontitis, Ji et al. [54,55,56] hypothesized the existence of different patterns of susceptibility among no periodontopathic, i.e., *Streptococcus*, *Actinomyces*, *and Veillonella*, and periodontopathic bacteria (Figure 7) to the major antibacterial defense mechanisms in the gingival sulcus, namely neutrophil-mediated phagocytosis and bactericidal action of two AMPs—LL-37 and β-defensin-3. In general, the results revealed a rather high variability across the species and an increased resistance to phagocytosis in the periodontopathic group but no significant differences in susceptibility to AMPs. However, more evident patterns were observed after splitting bacteria into the Socransky complexes, i.e., early colonizers (the yellow/green/purple complexes) versus the orange and red complexes as well as *A. actinomycetemcomitans*. Specifically, the members of the red complex (*P. gingivalis*, *T. forsythia*, and *T. denticola*) were recognized as the most resistant to both phagocytosis and LL-37. In Figure 7, we adapted the LL-37 susceptibility patterns from the study by Ji et al. [55] and supplemented them with MIC values from four other studies [57,58,59,60]. Indeed, the resistance to LL-37 in *P. gingivalis* has been recently connected with the major surface glycoproteins (Pgm6 and Pgm7), also called outer-membrane-protein-A-like proteins (OmpALPs) [61].

It should be noted that in the original paper linking periodontitis in patients with Kostman’s disease with *A. actinomycetemcomitans* [62], the authors reasoned its susceptibility to LL-37 based on a 3–4 log reduction in colony counts after 90 min treatment of 10^5^ bacteria with 20 μg/mL of LL-37 in 10 mmol/L phosphate buffer at pH = 7.2 with 1% yeast extract (TSBY). The methodology was derived from Tanaka et al. [63], who also reported the susceptibility of three tested *A. actinomycetemcomitans* strains, based on a 99% effective dose (ED_99_), i.e., the theoretical concentration of LL-37 at which there is a two log_10_ decrease in survivors after 1 h incubation with LL-37 at 37 °C, ranging from 8.2 to 11.6 μg/mL. Accordingly, substantial discrepancies between the MIC values for the same *A. actinomycetemcomitans* strains are reported by various studies, e.g., 62.5 vs. >200 (strain Y4) [55,58,59], 100 vs. >200 μg/mL (strain Y4) [55], and 50 vs. >200 μg/mL (strain ATCC 29523) [58,59]. Furthermore, susceptibility of three *T. denticola* strains to LL-37 was assessed by Rosenfeld et al. based on 85% growth inhibition following 1 h of exposure of 10^8^ bacteria to LL-37 at 50 μg/mL [64], which correlates with 62.5 μg/mL MIC value for this species by Ji et al. [55]. Remarkably, these values are in contrast with high LL-37 MICs (449.4 μg/mL) for two other spirochaetes—*Borrelia* and *T. pallidum* [65]. Overall, these discrepancies impede drawing definite conclusions regarding the role of direct bactericidal action of LL-37 in the eradication of the leading periodontopathogens.

**Figure 8:**
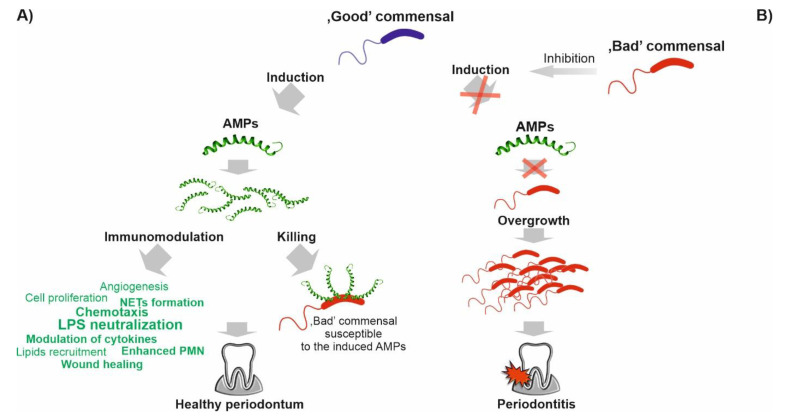
Scenarios of interactions between the oral bacteria and LL-37 and other AMPs. (i) “Good” commensals induce AMPs (and are resistant to the induced AMPs), while “bad” commensals are susceptible to the induced AMPs; (ii) “good” commensals, by inducing AMPs, enable the host to protect themselves from potential attack by pathogenic bacteria and to control eubiosis (panel **A**); (iii) “bad” commensals inhibit the pro-AMPs action of “good” commensals (panel **B**), adapted from [16].

Conversely, the importance of LL-37 in *A. actinomycetemcomitans* eradiation should be analyzed from a perspective of stimulation of LL-37 expression by vitamin D and synergistic action with responses mounted by TREM-1 as reported by McMahon et al. [17]. Briefly, the authors using a 3D air–liquid interface culture system observed that OKF6/TERT cells stimulated with 1,25(OH)2D3 for 24 h exhibit a significant increase in antibacterial activity against *A. actinomycetemcomitans* cells [17]. Accordingly, other immune responses promoted by LL-37, such as agglutination of *A. actinomycetemcomitans* and autophagy of *P. gingivalis*, might be responsible for their eradication [60,66]. Interestingly, the susceptibility of the oral bacteria to LL-37 has been recently connected with their oxygen requirements [46]. In detail, facultatively anerobic bacteria such as *S. mutans, S. sanguinis*, or *Actinomyces naeslundii* appear to be less susceptible to LL-37 than the obligate anerobic species, e.g., *F. nucleatum, Veillonella parvula*, and *Parvimonas micra*. Hence, oxygen may represent a selective agent for LL-37-mediated killing. Furthermore, the authors reported concentration-dependent dynamics of planktonic growth inhibition characterized by two steps: (i) primary—requiring lower LL-37 concentration (25–100 μg/mL), followed by (ii) a second significant reduction in growth at 100 mg/mL (or 250 μg/mL for biofilms) of both groups of bacteria—denoted as “threshold concentration.” Conversely, resistance to LL-37 may reflect an extraordinary “commensal” role of certain species, e.g., *F. nucleatum*, that due to the binding ability with other species, i.e., multigeneric coaggregation, is traditionally considered a “bridge” between the early and late colonizers [67]. In line with this, recent studies have shown that the *F. nucleatum* inducing expression of IL-8 and multiple AMPs may protect the host from “bad” commensals or non-beneficial/pathogenic bacteria [16]. For instance, *F. nucleatum subsp. nucleatum* (strain ATCC 25586 and 23726) and *F. nucleatum* subsp. *vincentii* (ATCC 49256) as naturally resistant to β-defensin 2 and 3, via inducing the expression of β-defensin 2 in oral epithelial cells through TLR-1/2 and TLR-2/6, along with LL-37 and CCL20, may act as homeostatic agents, ensuring protection against the “red” pathogen, *P. gingivalis,* which is susceptible to this AMP (and cannot induce its expression) [16,68]. In contrast, a poor induction of β-defensin 2 was noted in oral epithelial cells sensitive to *F. nucleatum* subsp. *polymorphum* ATCC 10953. Additionally, Ji et al. described *F. nucleatum* (and *P. intermedia*) as “self-limiting” commensals, i.e., inducing the host response that eliminates them efficiently and prevents overgrowth [54], which is consistent with its role in keeping the epithelium in a “heightened state of readiness” without promoting notable inflammatory cytokine responses [16]. Notably, since this protective action is mediated via cell-wall-associated FAD-I protein (Fusobacterium-Associated Defensin Inducer), it has been proposed as a novel, relying on the stimulation of AMP expression, immunoregulatory therapeutic agent to treat dysbiotic conditions in the oral cavity [16,68]. Importantly, recently, the ability of *F. nucleatum* to promote the expression of LL-37 in the presence of vitamin D3 has been also identified [16]. Considered as a “good” commensal, the *S. gordonii* M5 strain upregulates the expression of LL-37 and β-defensin-3 and is highly resistant to both AMPs (MIC > 125μg/mL) [55]. Interestingly, *S. gordonii* is also an essential prerequisite for further *P. gingivalis* colonization [67]. However, *P. gingivalis* can diminish these beneficial activities by downregulating the expression of proinflammatory IL-8 in the host cells, causing the so-called chemokine paralysis [69], as well as the inhibition of IL-8 activation by *F. nucleatum* and other “good” commensal. However, the mechanism of this process remains to be elucidated [16]. It is reported that the induction of IL-8 by such “good” commensals is responsible for the constitutive gradient in normal oral mucosa that contributes to the entry of percolating PMNs into the oral mucosa. On the contrary, Bachrach et al. suggested an opposite relation between *F. nucleatum* and *P. gingivalis*, i.e., the protection of *F. nucleatum* against LL-37 killing via its degradation by *P. gingivalis* protease (gingipain), as an example of a “group protection” mechanism [44,70]. Moreover, proteases of periodontal pathogens due to the degradation of the host protease inhibitors (SLPI and elafin) and, in turn, the stimulation of the protease 3 release may be considered direct modulators of LL-37 processing [71]. It is also noteworthy that certain pathogenic bacteria can use LL-37 as a gene expression inducer to promptly adapt to new conditions. For example, LL-37 upregulates the virulence genes of *Streptococcus pyogenes* via binding with its CsrRS receptor [72]. Therefore, different scenarios of interactions between the oral bacteria and LL-37 and other AMPs have been proposed (Figure 8) [16]. Scheb-Wetzel et al. showed that *E. faecalis* is highly susceptible to the antimicrobial effect of cathelicidin LL-37, which has been suggested in other studies as well [73]. In addition to its antibacterial activity, LL-37 inhibits *C. albicans* adhesion and growth. LL-37 can interact with the cell surface of *C. albicans* through its binding to cell wall polysaccharides, especially mannans, as well as exoglucanase Xog1. Notably, LL-37 causes cell aggregation, cell wall remodeling, and β-glucan exposure in *C. albicans* cells. Consequently, LL-37 strongly reduces *C. albicans* adhesion to plastic surfaces and oral epidermoid OECM-1 cells. LL-37 induces cell wall stress and the UPR, and Sfp1 contributes to the regulation of these stress responses [74].

It is worth also underlining that oral epithelial cells represent the first line of defense against viruses that are spread via saliva, including KSHV, herpesviruses (such as herpes simplex virus type 1), human cytomegalovirus, Epstein–Barr virus, and HPV. LL-37 might perform antiviral activities against those microorganisms due to its cationic nature and amphipathic structure, leading to binding to microorganism surfaces and subsequent virion perturbation [75]. LL-37, after virus envelope/capsid disruption, gains the ability to irreversibly bind to the virus DNA or RNA. LL-37 specifically has also been shown to enhance other methods of innate antiviral immunity, namely interferon expression. The analysis of docking studies and the display of positive interfacial hydrophobicity of LL-37 resulting in the disruption of COVID-19 viral membrane elucidate the fact that LL-37 could be effective against all variants of SARS-CoV-2. [76]. Overall, the induction of endogenously expressed LL-37 in oral epithelial cells may help to restrict oral infection.

## 4. LL-37 Peptide in Dental Caries, Pulpitis, Refractory Apical Periodontitis, and Periodontal Diseases

The oral cavity has a unique environment—hard tissues are not completely covered with epithelium but pierce through it. Such anatomy creates a specific environment that is constantly exposed to bacteria and associated infections that might take place within the tooth and the epithelium/junctional epithelium [77]. Periodontitis, pulpitis, periapical periodontitis, and caries are the most common diseases of the oral cavity. All of them have the features of opportunistic infections, which depend on biofilm formation and external factors, predisposing and modifying this process by changes in the host’s defense system. Periodontitis concerns the tissues supporting the tooth, i.e., the periodontium. These include the gums, bones, periodontal ligaments, and root cementum. The disease begins with the gums and is called gingivitis—gum disease disappears after the triggers are removed but, if left untreated, gingivitis turns into periodontitis, in which we observe irreversible changes in the periodontium—loss of connective tissue and bone. The progression of the disease leads to the mobility and subsequent loss of the tooth [46,78]. The main bacteria associated with periodontitis development are *P. gingivalis*, *T. denticola*, *T. forsythia*, and *A. actinomycetemcomitans*. These microorganisms have different virulence factors, such as bacterial collagenases, sulfides, and endotoxins (lipopolysaccharides). Moreover, *A. actinomycetemcomitans* releases leukotoxin, while *P. gingivalis*, *T. denticola*, and *T. forsythia*, release trypsin-like peptidases. Regardless of the biofilm, the occurrence and course of periodontitis may be influenced by modifying and predisposing factors such as dry mouth, biofilm retention factors, smoking, metabolic factors, poor nutrition, medications, various systemic diseases (e.g., hematological disorders and endocrinological disorders (diabetes)), and stress. Periodontitis, a chronic inflammatory disease, occurs when the balance between the patient’s immune system and the pathogenicity of the bacteria is upset. Its development depends on whether the immune system (neutrophils) complements and antibodies neutralize the metabolites of the bacterial plaque and its metabolites in the periodontal connective tissue. Neutrophils prevent bacteria and their metabolic products from getting into the connective tissue, but if they get there, pro-inflammatory cytokines IL-1 and PGE2 are released, which increase inflammation and thus the destruction of connective tissue (including bones). Periodontal pockets are formed, which provides even easier conditions for the development of periopathogens [79]. In 2017, a new periodontal classification was created. The European Federation and the American Academy of Periodontology have established based on pathophysiology that there are three types of periodontal disease, periapical periodontitis, peri-implantitis, and periodontitis, characterized by inflammation and the destruction of alveolar bone and connective tissues.

Caries represent a dysbiotic state of the oral microbiota related to the frequent consumption of sugars, poor hygiene, and bad patient compliance. *S. mutans* is a human cariogenic pathogen that can ferment sugar into lactic acid, acidifying the environment and promoting the development of acid-resistant bacteria and the subsequent disease. This disease refers to hard teeth tissue and begins with the demineralization of the enamel. The progression of the disease causes the breakdown of dentin (the tissue under the enamel), followed by the involvement of the dental pulp filling the tooth chamber and its death. Caries treatment consists of preventing bacterial plaque deposition at an early stage; in the case of enamel involvement conservative treatment by filling the cavity with composite material; and when reaching the dentin or pulp, using materials that stimulate the cells of these tissues to regenerate the dentin pulp complex, i.e., for the production of repair dentin known as the dentine bridge [80,81,82]. According to research by Davidopoulou, LL-37 is present in saliva in early childhood and its concentration increases with age to achieve equilibrium in maturity. Among others, the concentration of cathelicidin in saliva depends on milk consumption, age, number of teeth, and various mechanical stimuli [83,84,85]. The expression of LL-37 in unstimulated saliva in children with a high caries index is low compared to similar age patients with average caries activity. In general, the LL-37 concentration is significantly higher in patients without and with low caries activity [58,83]. In addition, the protective role of LL-37 in dental caries was suggested in patients with edentulism [86]. However, the opposite results were presented by Colombo et al. [87], who observed that only combinations of LL-37 with β-defensins and histatin-5, but not LL-37 alone, were positively associated with the caries levels. For example, Phattarataratip et al. observed that *S. mutans* strains isolated from caries-free patients were more susceptible to LL-37 than those isolated from caries-active subjects, though without statistical significance [88]. It is worth underlining that LL-37 achieves an antibiofilm effect by reducing the adherence of bacteria to the tooth surface, as well as by interfering with the thickness of the biofilm. Moreover, conventional antibiotic therapy usually is not effective in eliminating biofilm bacteria, mainly due to the high resistance of bacteria to antibiotics and the incompetence of antibiotics to penetrate the biofilm, while LL-37 handles those difficulties. The direct bactericidal effect is based on the lysis of bacteria and the inhibition of the pro-inflammatory activity of bacteria wall components, especially LPS and teichoic acid. As a result, the production of cytokines (IL-1, IL-6, and TNF-alfa) and other inflammatory responses is reduced. Importantly, LL-37 provides the maximum inhibitory effect on primary colonizers, i.e., the yellow–orange complex, whereas the pathogenic red complex is resistant to the LL-37, as mentioned above. Moreover, some bacteria can produce enzymes, mostly proteases, that inactivate LL-37. *P. gingivalis* can produce arginine-specific gingipains, cysteine proteases that inactivate the LL-37 peptide (arg-gingipains and lys-gingipains) [44]. Similar proteases are secreted by the red complex periodontal pathogens [89]. *T. forsythia* releases various MMP-carinase, which use LL-37 as a substrate. Mirolase, a subtilisin-like serine protease, degrades cathelicidin [90]. LL-37 might also behave similar to an opsonin because it facilitates the removal of antigens by the immune system. Apart from these actions, LL-37 directly affects fibroblasts (cells with the greatest number in periodontal tissues)—it enables their migration to the site of inflammation [91] and stimulates the release of IL-8, IL-6, and TIMP-1, as well as beta-FGF, HGF, and KGF [91], which may stimulate the regeneration of periodontal tissues and thus the remission of the disease [92]. Overall, the impairing protective role of LL-37 in maintaining the oral health stability by direct (e.g., enzymatic degradation by bacterial proteases) and indirect factors related to dysbiotic conditions is responsible for the development and progression of caries.

**Figure 9:**
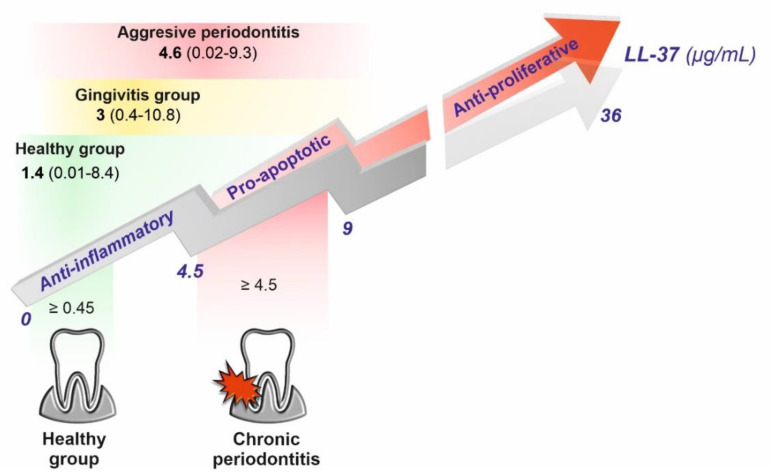
Illustration of concentration-dependent anti-inflammatory (exerted at a concentration ≥ 0.45 μg/mL or 0.1 μM), pro-apoptotic (exerted at a concentration ≥ 4.5 μg/mL or 1 μM), and anti-proliferative (exerted at a concentration ≥ 36 μg/mL or 8 μM) activity of LL-37 in gingival crevicular fluid (GCF) postulated by Jonsson at al. [95]. The authors correlated the anti-inflammatory and pro-apoptotic values with concentrations of LL-37 in GCF patients with chronic periodontitis and the healthy control group from the reference [105]—the lower part of the image (the size of areas highlighted in green and red represent the upper and lower quartiles of LL-37 concentrations in the healthy and chronic periodontitis group, respectively). The upper part of the image shows LL-37 concentrations in GCF patients with gingivitis and aggressive periodontitis in comparison to the healthy control group from the reference [45] (the size areas of highlighted in green, yellow, and red and the higher color intensity represent the range of LL-37 concentrations (the values in parentheses) and the means, respectively).

Chronic apical periodontitis is a lesion formed by the periradicular host defense as a response to microorganisms present in the root canal system. Although no specific microorganism has been identified as the principal etiologic agent of pulpal and periapical pathosis, some species have been more frequently reported in the root canal space. Previous studies have shown that such species as *E. faecalis*, *Eubacterium*, *Fusobacterium*, *Peptostreptococcus*, *Porphyromonas*, *Prevotella*, and *C. albicans* are commonly encountered in endodontic infection [93,94]. Interestingly, Jonsson et al. observed that the treatment of periodontal ligament cells with LL-37 in the range 0.1-1 µM completely inhibited the expression of LPS-induced monocyte chemoattractant protein-1 (MCP-1) and reduced the production of interleukin-6 by 50–70% [95]. On the contrary, pro-apoptotic activity, reflected by elevated levels of caspase 3 (an apoptosis mediator), required 1 µM doses of LL-37, whereas anti-proliferative effects were exerted at an 8 µM concentration of LL-37. Remarkably, the obtained values are well correlated with levels of LL-37 in GCF patients with chronic and aggressive periodontitis (Figure 9). Another study illustrates the dual, concentration-dependent, nature of LL-37, that on crossing a certain threshold, instead of promoting tissue and bone regeneration or wound healing, contributes to their destruction [96].

In addition to the above-reported discoveries, LL-37 is characterized by anti-osteoclast properties. As evidenced, cathelicidin LL-37 limits bone resorption by inhibiting TLR ligand and reduces LPS-promoted osteoclast production, as well as stimulates angiogenesis and bone regeneration. Moreover, LL-37 inhibits LPS-induced inflammation, promotes BMSC proliferation and migration, and stimulates the osteogenic differentiation of BMSCs in both normal and inflammatory microenvironments via the P2X7 receptor and MAPK signaling pathway [97]. Interestingly, it was recently reported that osteo-inductive biomaterials regulate gene expression through the MAPK pathway. They are used in periodontal microsurgery to regenerate periodontal tissue [97]. Accordingly, osteogenic differentiation and increase in calcium mineral deposition mediated by LL-37 were demonstrated by Liu et al. [98]. The LL-37-linked differentiation processes are also crucial for the continuity of permanent teeth development when immature teeth suffer from periodontitis or progressive carious lesions and thus pulpal necrosis occurs. In such cases, LL-37 was reported to stimulate migration and odonto/osteogenic differentiation of stem cells from the apical papilla (SCAPs), being the main source of cells for the primary dentin formation in the roots and seed cells for pulp regeneration, through the Akt/Wnt/b-catenin signaling pathway [99]. Recently, there has been a lot of research on the regenerative treatment of the dental pulp, which would reduce the number of root canal treatments and increase the prognosis for long-term maintenance of the teeth in the oral cavity. LL-37 can stimulate odontoblastic cells to form reparative dentin, as well as fibroblast-like stem cells that are multipotent and are likely to polarize, among others, to odontoblasts or osteoblasts; nevertheless, the whole mechanism requires further study. It is believed that cathelicidin LL-37 will benefit as a pulp capping agent as the forerunner for cell differentiation and the creation of a dentin bridge [100,101,102]. The LL-37-mediated increase in dentine sialophosphoprotein (DSPP) production and *DSPP* gene expression contributes possibly to the differentiation of dental pulp stem cells into odontoblast-like cells [103]. Cathelicidin-related antimicrobial peptide (CRAMP, encoded by the Camp gene) was found to be expressed in rat odontoblasts at the early dentinogenesis stage, and formyl peptide receptor 2 (FPR2), i.e., one of the transmembrane G-protein-coupled receptors to which CRAMP/LL-37 binds to cause various physiological effects, is constantly expressed in the sub-odontoblastic layer. Additionally, both CRAMP and FPR2 were recorded to be expressed in response to cavity formation, indicating that both of these agents appear during physiological and reparative dentin formation [104]. Moreover, in the report by Kajiya et al., LL-37 was demonstrated to enhance the migration of human pulp cells and thus increase pulp–dentin complex regeneration by the activation of epidermal growth factor receptor (RGFR) and c-Jun N-terminal kinase by the induction of heparin-binding cell migration [100].

As widely recognized, the inflammatory environment and vitamin D intensify the production of the LL-37 peptide [106]. Furthermore, McMahon et al. have observed that due to the endogenous expression of 1-α-hydroxylase in the gingival epithelial cells, systemic vitamin D treatment may be sufficient to activate the innate immune response in these cells [17]. Interestingly, a strong association of the VDR gen polymorphism with chronic periodontitis has been revealed by a recent meta-analysis [107]. Recently, triggering TREM-1 has also been proposed as a potential target for the treatment or prevention of oral cavity diseases. Indeed, several other studies have underlined the importance of TREM-1 in periodontal diseases [108,109,110]. For instance, increased TREM-1 expression in gingival tissue was observed in both chronic and aggressive periodontitis and correlated with the levels of the red complex species, whereas a significantly elevated load of *A. actinomycetemcomitans* was noted only in the aggressive form [111].

**Figure 10:**
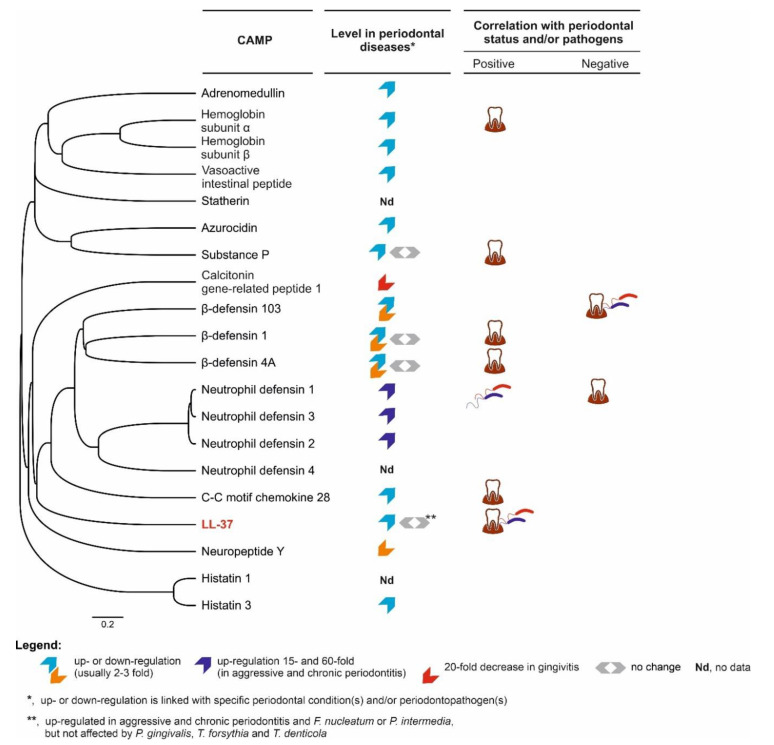
Comparison of 20 human cationic antimicrobial peptides (CAMPs) present in oral cavity, including LL-37 (highlighted in red), based on the amino acid sequence similarity as well as changes in their expression in periodontal conditions and correlation with the periodontal status and/or bacterial pathogens. The alignment of hCAMP sequences (only aa representing active peptides were aligned, i.e., without signal sequences and/or proteolytically cleaved fragments) and their phylogenetic tree was performed using MAFFT version 7 server (based on the L-INS-I alignment algorithm and average linkage—UPGMA, respectively) [112]. The data regarding the hCAMP expression levels (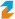, i.e., up- or downregulation) and their correlation with the periodontal status (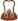, represented by probing depth, PD; bleeding on probing, BOP; clinical attachment loss, CAL; plaque index, PI; gingival index, GI; papillary bleeding index, PBI) and microbiological parameters (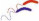, i.e., changes in occurrence of *P. gingivalis*, *T. forsythia*, and *T. denticola*) were obtained from the references [5,6,113].

It was evidenced that patients susceptible to caries and children with high carious activity have lower levels of LL-3 and even a lack of peptide might be observed in patients with some neutrophil deficiency or Kostmann syndrome, Papillon–Lefèvre (PLS) syndrome, or Haim–Munk syndrome (Figure 10) [62,113,114,115]. A lack or impaired proteolytic processing of hCAP-18 is observed in patients with Papillon–Lefèvre and Haim–Munk syndromes, caused by the mutational inactivation of the cathepsin C gene. In turn, the reduced activation of neutrophil serine proteases, i.e., proteinase-3 results in the development of severe periodontal disease [115,116]. Interestingly, the periodontal pathogens may exert the opposite action, i.e., increase the level of the proteinase 3 [71]. To confirm, significantly elevated levels of the proteinase 3 in GCF samples from patients with periodontitis and gingivitis, along with a positive correlation with clinical periodontal parameters of sampling sites, have been reported [117]. To sum up, LL-37 is responsible for limiting the periodontal and caries pathogens and inhibiting the progression of periodontal disease. LL-37 might also act as a diagnostic tool (early marker of inflamed tissues), a prognostic tool in periodontitis, and also a potential agent for the prevention and treatment of periodontal diseases.

## 5. LL-37 Peptide in Diseases of Oral Mucosa and Its Implication in Oral Cancer Development

Some of the pathogens responsible for periodontal and pulp diseases, as well as mucosa diseases, may also be the etiological factors of pre-cancerous conditions and oral cavity cancers, e.g., *P. gingivalis* or *C. albicans* were isolated from the keratinocytes of patients with squamous cell carcinoma of the oral cavity [118]. The participation of yeasts in the process of oncogenesis has been known for a long time, and, in part, it was related to the *Candida* ability to produce nitrosamine [119]. Interestingly the mutual interaction between *P. gingivalis* and *C. albicans* might be of importance since *P. gingivalis* causes the formation of elongated hyphae in *C. albicans*, while the fungus increases the expression of genes responsible for the virulence of this periopathogen [119,120]. In this setting, LL-37 may prevent the formation of neoplastic chambers by promoting *P. gingivalis* autophagy in keratinocytes and influencing TRIM22 and LAMP3 [66]. As for *C. albicans*, LL-37 influences cell wall remodeling, reducing adhesion of *C. albicans* to the surface cathelicidin, which can bind to mannans and Xog1 exoglucanase, stimulating the Mkc1 MAP kinase pathway to maintain wall integrity [74]. *Candida’*s epithelial invasion can cause hyperplastic conditions probably only when the infection is chronic, deep, and associated with risk factors such as tobacco and alcohol, or others [119]. Moreover, *F. nucleatum* has been implicated in oral cancers since enriched fusobacterial organisms have been found in leukoplakic lesions; i.e., precursors of OSCC [121]. Oral ulcers, one of the clinical conditions representing a sign of Behcet’s disease (BD) associated with cancer development, are linked to the adhesion of *S. sanguis* to buccal epithelial cells. A study by Mumcu et al. has shown a positive correlation between salivary levels of LL-37 and the number of monthly oral ulcers in patients with Behcet’s disease [122].

Importantly, altered expression of hCAP18/LL-37 was observed in oral inflammatory lesions with and without microbial infection or oral cancer. Noteworthy, inflammatory conditions in the oral mucosa might raise the concentration of LL-37 in saliva, which might directly promote carcinogenesis, considering that inflammation has now been confirmed as the seventh hallmark of tumors. One observation that links LL-37 ability to cancer development is the LL-37 ability to stimulate cell proliferation by decreasing cell apoptosis [123]. LL-37 was also reported to be engaged in oral submucous fibrosis (OSF) development, which is a chronic disease significantly contributing to mortality since a high malignant transformation rate was observed in subjects that were diagnosed with this condition (1.5–15%) [124]. Pathological characteristics of OSF include chronic inflammation, excessive collagen deposition in the connective tissues below the oral mucosal epithelium, local inflammation in the lamina propria or deep connective tissues, and degenerative changes. Causative factors of OSF include autoimmunity; vitamins B, C, and iron deficiencies; chewing betel nut; consumption of spicy foods; human papillomavirus (HPV) infection; and genetic mutations [124]. Oral lichen planus (OLP) is another chronic inflammatory disease of the oral mucosa that might lead to changes in LL-37 expression, but its etiology is unknown. It is characterized by the lysis of the basal keratinocytes after being damaged by cell-mediated immune reactions [125]. In patients with OLP, the salivary concentration of LL-37 was significantly higher than in healthy subjects. The oral lichen planus (OLP) expresses more hCAP18/LL-37 peptide, as detected by intense immunohistochemical staining, compared to the healthy epithelium, and this increased expression is not related to microbial infections [126]. Since it is estimated that 1 in 6 cancers in the oral cavity is attributable to either viral or bacterial infections, the positive loop between infection, inflammation that manifests by host increase of hCAP-18/LL-37 expression, and oral tumor development should be considered as the molecular background of many different malignancies. The molecular nature of different microorganism might be of importance as well, since some bacteria are considered more likely to be involved in oncogenesis, as mentioned above.

To date, LL-37 has been established to display variable, tumor-biology-dependent effects on cancerous cells, both pro- and antitumorigenic [127]. As demonstrated in a broad spectrum of studies, LL-37 suppresses colon and gastric cancer progression, as well as exerts anti-cancer effects against hematological malignancies via the molecular mechanisms that involve triggering of caspase-independent apoptosis, a decrease in proteasome activity, and inhibition of angiogenesis processes by FPR1 activation [128,129,130]. At the same time, by recruiting MSCs and increasing their invasiveness, activating EGFR and MEK/ERK1/2 signaling pathways, as well as stimulating ERB-family receptors, human cathelicidin acts as a tumor promoter supporting the invasive phenotype of cancer cells [131,132,133]. For this reason, the role of LL-37 in cancer expansion is not clearly defined and unified for all cancers, since its effect is tissue specific. In the aspect of oral cavity tissues, the amount of data on LL-37′s impact is unsatisfactory to date. CAMP/LL-37 expression is significantly downregulated in OSCC, and its low expression correlates with histological differentiation and lymph node metastasis. More importantly, a cell-specific methylation pattern in the promoter region of human CAMP was detected, which suggests that LL-37 plays a suppressive role in the progression of this cancer [134]. Indeed, LL-37 was reported to trigger caspase-dependent apoptosis by the P53-Bcl-2/BAX signaling pathway in the human OSCC HSC-3 cells [135]. In another study, the anti-tumor effects of the synthetic fragment of the LL-37 peptide, KI-21-3, against oral squamous cell carcinoma were demonstrated in in vivo settings and concluded as resulting from antiproliferative and proapoptotic activities of this compound [136]. Similarly, the C-terminal domain of human CAP18(109–135) was reported to induce mitochondrial depolarization and apoptosis in tongue squamous cell carcinoma SAS-H1 cells [137]. Importantly, the cytotoxic effect was not reproduced in healthy human gingival fibroblasts (HGF) and human keratinocyte line (HaCaT) [137], which could be attributed to the different structure and physicochemical features of cancerous cells when compared to the healthy ones (such as the presence of cholesterol-rich lipid rafts or different surface charge) [138]. Nevertheless, it was not characterized in detail. In contrast to these promising reports, a comparative analysis of the biological effects of LL-37 against three oral tongue squamous cell carcinomas revealed its fluctuating effect and variable impact on malignant phenotype. Although exogenous LL-37 decreased mostly proliferation of cancer cells, it stimulated their migration and invasion at the same time, as well as promoted MMP-2 and MMP-9 expression, suggesting a pro-tumorigenic effect. Interestingly, it enhanced the total amount of EGFR, but the EGFR pathways were recorded to be mostly decreased. Finally, the expression of hCAP18/LL-37 decreased with the severity of oral dysplasia and was lower in cancer tissue samples, but it did not correlate with the clinical outcomes of the patients [139]. These data suggest that determining the exact role of LL-37 in the development of oral cancers may be hampered due to the difficulty in correlating the expression of this peptide with the aggressiveness of a given cancer. Certainly, the LL-37 peptide shows many beneficial effects in protecting oral tissues from malignant transformation—one of the most relevant reports on this aspect is the study by Brice et al., indicating the restriction of Kaposi’s sarcoma-associated herpesvirus (KSHV) infectivity, which results from direct disruption by LL-37 of the viral envelope, which inhibits its entry into oral epithelial cells, limiting ultimately KSHV-associated disease occurrence [140]. The crucial role of anti-inflammatory properties of LL-37 should also be recognized considering the ever-increasing number of reports linking the severity of inflammation in tissues to the development of tumors within them [141]. Nevertheless, given the multifactorial nature of cancer transformation and the spectrum of factors that influence the invasiveness of cancer tumors, some caution is needed in determining the true role of this peptide in this aspect of oral health. However, considering the pleiotropic biological activities of LL-37 resulting from the ability to interact with a spectrum of different membrane receptors, the exploration of the potential effect of this peptide on the development of cancers in the oral cavity as well is justified.

## 6. LL-37 Peptide as a Guardian of Oral Mucosa Mechanical Properties (LL-37 in Saliva and Saliva–Mucosal Surface Interference)

**Figure 11:**
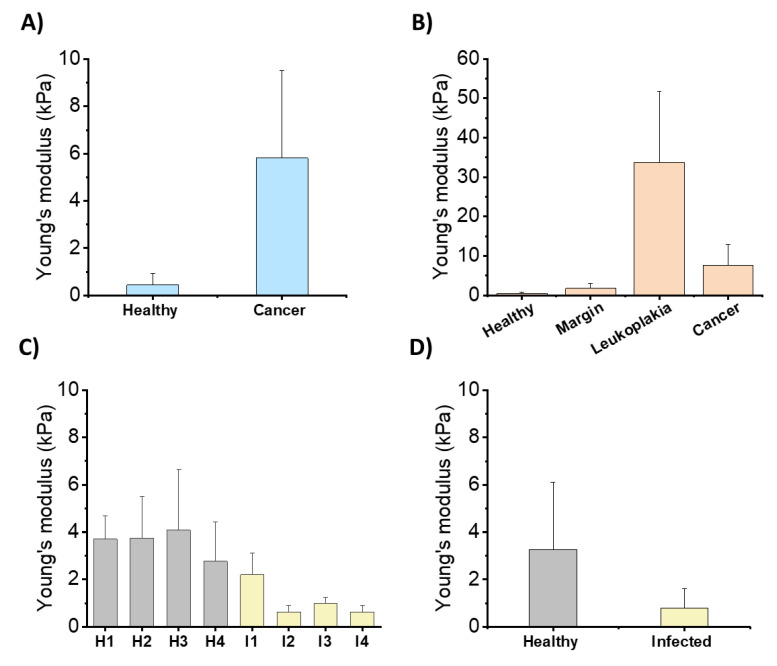
Young’s modulus values obtained for healthy and diseased tissues using the AFM indentation technique: (**A**) Average Young’s modulus values of healthy and colon cancer tissues. A significantly higher Young’s modulus of neoplastic tissues as compared to healthy tissues was shown. The greater stiffness of neoplastic tissues is associated with the presence of an increased amount of extracellular matrix protein. Collagen overexpression, matrix fibrosis, cross-linking, and vascularization occur during tumor progression [146]. (**B**) Young’s modulus value distribution for healthy mouth mucosa and diseased tissues (leukoplakia and cancer). Differences between healthy tissue and tissue outside of the leukoplakia area are noticed. The stiffness of the leukoplakia samples was higher compared to the surrounding mucosa. Inhomogeneity of stiffness within leukoplakia samples was observed, which might act as a mechano-agonist that promotes oncogenesis. Stiffness of cancer samples was significantly lower than that within the precancerous ones [152]. (**C**) Mechanical properties of healthy stomach tissues (H1-H4) and those infected with *Helicobacter pylori* (I1-I4). The mean values of tissues’ Young’s modulus ± standard deviation for each healthy and infected tissue [147]. (**D**) Rheological difference between healthy stomach tissue and tissue during inflammation caused by *H. pylori* infection. The mean values of tissues’ Young’s modulus ± standard deviation [147].

In recent years, new evidence has indicated that the dysfunction of physiological processes during infection, development of inflammation, and carcinogenesis generate structural changes in cells, cellular organelles, and tissues. These alterations translate into changes in the rheological properties of these biological structures. [142,143,144,145,146,147,148]. New techniques that allow us to measure the rheological properties of biological samples (cells and tissues) have made it possible to determine the rheological properties of different biological structures. [143,145,149]. Recent work has also shown the modifications in the mechanical properties of tissues within the oral cavity due to pathological changes [150]. Some lesions, such as condylomas, and focal epithelial hyperplasia are at least in part associated with HPV infections. If left undiagnosed and untreated, they might lead to cancers [151]. Changes in tissue structures can be visible using microscopic observations, but some of these changes can only manifest themselves as changes in tissue mechanical properties, which nowadays are often described as mechanomarkers. Interestingly, some mechanical changes can be observed earlier than changes in standard histopathological examinations [146] (Figure 11).

Since the first in vitro observation indicating the ability of the LL-37 peptide to increase the cell stiffness of epithelial cells, the new mechanism of LL-37 action might also be based on its ability to modulate viscoelastic properties of the cells and tissues. It has been shown that changes in cell and tissue mechanics under the influence of chronic microbial colonization can, in the long term, disrupt transcriptional processes in the cell nuclei of epithelial cells and thus cause cancer risk [147,153]. Studies of gastric tissues from children with confirmed *H. pylori*-induced inflammation indicated that the infected tissues had lower stiffness compared to healthy tissues. Since *H. pylori* can colonize the oral cavity, a similar effect on oral tissue as the response of tissue mechanics during the inflammatory process might be expected. Interestingly, some changes in tissue’s mechanical properties might be mediated by *H. pylori’s* virulence factors [147]. It is tempting to assume that within the oral cavity, we will also observe similar changes. Panel 1B shows the results of extracellular matrix stiffness within leukoplakia of the human oral mucosa, which as previously mentioned here is known for the increased expression of hCAP-18/LL-37. In our study, we observed heterogeneity in stiffness within leukoplakia samples, reflecting an increase in collagen regeneration and accumulation (increasing density) in the extracellular matrix (ECM) and an increase in the stiffness of leukoplakia samples compared to the surrounding mucus. These results indicate that the changes occurring also in the oral cavity under the influence of pathological processes, inflammatory or neoplastic conditions, change the rheological properties of tissues. The influence of saliva components, including LL-37, on the mucosa plays an important role in these processes. As already mentioned here, saliva rich in antimicrobial peptides, including LL-37, plays a major role in the pathophysiology of mucosa, representing a natural barrier of the oral cavity [83,154,155]. In addition to LL-37, saliva contains a number of components with different actions, including histatin, a polypeptide with antifungal and antimicrobial activities; mucins, a group of glycoproteins that contribute to the viscoelastic nature of mucosal secretions; proline-rich proteins (PRPs); and other salivary proteins, such as cystatins, amylase, and kallikrein. In addition, saliva contains proteins such as lysozyme and secretory immunoglobulin A (sIgA), which are also part of the immune system [156]. These saliva components interact to a greater or lesser extent with oral tissues as well as biomaterials that may be present in the oral cavity [157].

The LL-37 peptide, through its broad protective properties, may act as a guardian of the normal mechanics of oral tissues, influencing the pathological factors causing its mechanical changes (Figure 2). This is particularly important due to the fact that mechanical properties are essential for the proper functioning of the human body [147]. In previous work [157], the anti-inflammatory effect of LL-37 at the site of infection was associated with LL-37-mediated increase in HUVECs epithelial cell stiffness, which prevents increased pericellular permeability. This effect is associated with inflammation that can lead to local accumulation of tissue fluid. This homeostatic performance of the LL-37 peptide accounts for the stabilization of tissue mechanics at values that characterize healthy and normal tissue. Changes in cell stiffness in the presence of the LL-37 peptide [158] indicate that after adding LL-37, sphingosine-1-phosphate, and LPS or infection with *P. aeruginosa*, an increase in stiffness and F-actin content was observed in the cortical area of A549 cells and primary human lung epithelial cells. The LL-37-induced increase in cell stiffness was accompanied by a decrease in the permeability and uptake of *P. aeruginosa*. It can be concluded that the antibacterial activity of LL-37, in addition to its direct action on bacterial cells, involves the interaction between LL-37 and the multicomponent lipid membrane [30,36,158]. It consists in increasing the rigidity of the cells on which bacterial cells are deposited, which reduces the ability of bacteria to translocate into the epithelium [127]. The reduced ability of bacteria to invade protects tissues from changes in their mechanics, which in the long term may protect against more severe changes in tissue structure. It has been shown that changes in cell and tissue mechanics under the influence of chronic microbial colonization can, in the long term, disrupt transcriptional processes in the cell nuclei of epithelial cells and thus cause cancer risk [148,159].

## 7. Conclusions

**Figure 12:**
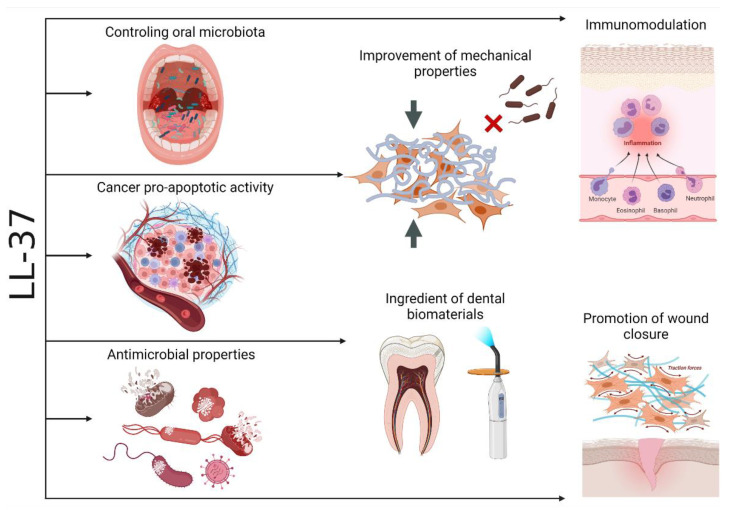
Cathelicidin LL-37 as a pivotal factor in maintaining homeostasis of the oral cavity.

The sum of the above-cited papers indicates that human cathelicidin LL-37 plays a crucial role in oral cavity homeostasis by the modulation of the inflammatory cytokine content and, thus, its cellular effects on dental tissues, providing a suitable microenvironment for vascularization, promoting the mesenchymal stem cells’ differentiation and migration, and limiting the effects of bacteria-derived inflammatory factors due to its bactericidal activities. In might be predicted that in the future, immobilization of the LL-37 peptide or its peptidomimetics in a resin composite filling or a polymerizable root canal sealer would achieve the purpose of using cationic peptides in combating some oral cavity pathologies, such as caries and pulpal infections (Figure 12).

## Data Availability

All sources of data reported are provided as references in figure descriptions.
